# Characterization and Evaluation of the Cytotoxicity of Pregabalin Gels for Oral Application

**DOI:** 10.3390/ph17091168

**Published:** 2024-09-04

**Authors:** Gabriela Monteiro Barbosa Xavier, Lindalva Maria de Meneses Costa Ferreira, Marcele Fonseca Passos, Ana Paula Drummond Rodrigues, Felipe Tuji de Castro Franco, Cecy Martins Silva, José Otávio Carréra Silva Júnior, Roseane Maria Ribeiro-Costa, Jesuína Lamartine Nogueira Araújo

**Affiliations:** 1 School of Dentistry, Federal University of Pará, Belém 66075-110, PA, Brazil; gabrielamontb@gmail.com (G.M.B.X.); cecymsilva@gmail.com (C.M.S.); 2 Laboratory of Pharmaceutical Nanotechnology, College of Pharmacy, Federal University of Pará, Belém 66075-110, PA, Brazil; lindalva.costa.ferreira@ics.ufpa.br (L.M.d.M.C.F.); roseribeiro01@yahoo.com.br (R.M.R.-C.); 3 Biotechnology School, Federal University of Pará, Belém 66075-110, PA, Brazil; cellepassos@gmail.com; 4 Electron Microscopy Laboratory, Evandro Chagas Institute, Belém 66093-020, PA, Brazil; ninhadrummond@gmail.com (A.P.D.R.); felipe.tuji@gmail.com (F.T.d.C.F.); 5 Laboratory of Pharmaceutical and Cosmetic R&D, College of Pharmacy, Federal University of Pará, Belém 66075-110, PA, Brazil; carrera@ufpa.br

**Keywords:** pregabalin, gel, formulation, cytotoxicity, gingival fibroblasts

## Abstract

The efficacy of pregabalin in pain treatment has led to the search for new formulations for its use through different routes of administration. This study aimed to prepare, characterize, and evaluate the cytotoxicity of pregabalin (PG) gels for topical application in the oral cavity. Solutions with three different concentrations of PG were prepared and added to a 1.0% carbopol gel base. Thermal analyses (TG and DSC) and FTIR were performed on the gel and pure pregabalin. Stability (preliminary and accelerated) and rheology studies were also conducted on the gels. Cytotoxicity was evaluated in human gingival fibroblasts in the following groups: WG (1.0% carbopol gel base), PG2G (2.0% pregabalin gel), PG5G (5.0% pregabalin gel), and PG10G (10% pregabalin gel). A transparent and homogeneous gel with a pH of 6 was obtained. The formulations showed stability, and the different drug concentrations did not influence the product’s characteristics. None of the tested groups showed cytotoxicity for the analyzed cells. The pregabalin gels exhibited favorable and non-toxic characteristics for human gingival fibroblasts in vitro. Therefore, this product may be a promising therapeutic alternative for topical application in the oral mucosa.

## 1. Introduction

The use of anticonvulsants in pain treatment has proven to be quite efficient due to their ability to reduce neural excitability [1]. Pregabalin (PG), a structural analog of gamma-aminobutyric acid (GABA), which acts in models of acute and inflammatory neural-origin pain, has been very effective in treating painful symptoms [2]. This drug reduces neuronal excitability in the central and peripheral nervous systems [3,4,5] by binding to the α2δ subunit of voltage-dependent calcium channels present in the presynaptic terminals of neurons [6].

Pregabalin has the potential to reduce Ca^2+^ influx and neurotransmitter release (substance P, glutamate, and CGRP), which is responsible for its analgesic and anticonvulsant action [2,7]. This Ca^2+^ influx reduction promoted by pregabalin occurs through the inhibition of VGCC and TRPV1 channels in various neurons [8]. Its mechanism of action also promotes anti-inflammatory effects, stemming from the blockade of α2δ-1 subunit expression and activation of the NF-κB pathway, with the latter being the main factor in the production of inflammatory mediators [3,5,9].

Several studies on neuropathic pain models demonstrate that systemic treatment with pregabalin can effectively and durably attenuate pain [10,11]. However, the use of this drug typically comes with a series of side effects, such as dizziness, drowsiness, mental confusion, and disorientation [12]. Such implications cause significant discomfort for the patient, making its use difficult, especially for those who are more debilitated or have other associated problems [13]. In light of this, formulations for topical application of pregabalin have been investigated and show promising and effective results [14,15].

The topical use of this drug in the form of gel, hydrogel, and organogel reveals new efficient delivery systems for its release and action [16,17]. Furthermore, they significantly reduce the likelihood of adverse effects, which are very common with oral use of pregabalin. Another point to highlight is that smaller doses can be used through the topical route of administration, and yet, the activity of pregabalin in reducing pain remains satisfactory [14,16,17,18]. Although previous studies demonstrate the effectiveness of topical PG application, all refer to formulations for action on the dermis, and there are no reports of investigations aimed at a pharmaceutical form suitable for application in the oral cavity.

Thus, despite the evaluation of pregabalin in different pain models and cell types, there are no reports of studies on pregabalin formulations for the oral cavity. In this context, in vitro cytotoxicity tests are reproducible options, ideal for predicting clinical outcomes and suitable for evaluating the basic biological properties of materials for dental and pharmaceutical use [19,20]. Additionally, a physicochemical evaluation of the product prior to its application can be used to predict its quality and safety. Therefore, this study aimed to obtain and characterize pregabalin gel formulations for topical application in the oral cavity and to evaluate cytotoxicity in human gingival fibroblasts. The experimental hypothesis is that the pregabalin gel will present appropriate physicochemical properties and biocompatibility with human gingival fibroblasts.

## 2. Results

### 2.1. Obtaining the Gel Formulation

After incorporating the solutions with different concentrations of pregabalin (PG2G, PG5G, and PG10G) into the 1.0% carbopol gel base, consistent and transparent gels with a homogeneous appearance were obtained.

### 2.2. Characterization of the Drug and Gel Formulation

#### 2.2.1. Fourier-Transform Infrared Spectrometry (FTIR)

**Figure 1:**
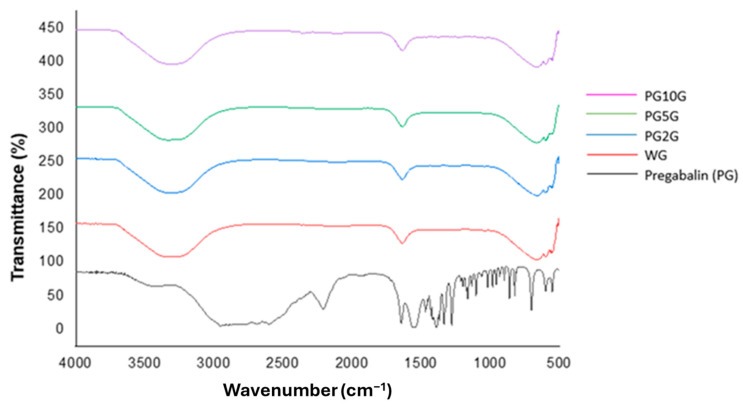
Spectra of pregabalin (PG) and the different gels in the infrared region (FTIR), namely, WG (1.0% carbopol white gel), PG2G (2.0% pregabalin gel), PG5G (5.0% pregabalin gel), and PG10G (10% pregabalin gel).

The spectrum of PG (Figure 1) exhibited peaks at 2947 cm^−1^ (C-H stretching), 1636 cm^−1^ (N-H bending), 1548 cm^−1^ (asymmetric stretching N-O), 1467 cm^−1^ (axial C-H bending), 1330 cm^−1^ (symmetric stretching N-O), 1276 cm^−1^ (C-O stretching), and 964 cm^−1^ (O-H bending). In the FTIR spectra of the base gel and PG gels, the most significant absorption bands can be observed at peaks of 3304 cm^−1^ (O-H) and at the peak of 1642 cm^−1^. The gel and pregabalin coincidentally have acute effects at the same wavelength; however, the peak of pregabalin is sharp and not as well-defined as the peak of the gel. The peak of the gels loaded with pregabalin at 1650 cm^−1^ is the same as the peak of the gel without PG.

#### 2.2.2. Thermogravimetric Analysis (TG/DTG)

**Figure 2:**
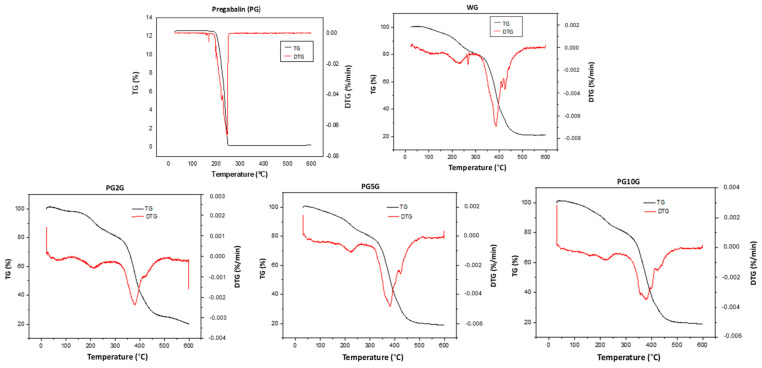
Thermogravimetric analysis (TG/DTG) of pregabalin (PG) and formulations, namely, WG (1.0% carbopol white gel), PG2G (2.0% pregabalin gel), PG5G (5.0% pregabalin gel), and PG10G (10% pregabalin gel).

The drug mass loss occurred in two different stages. Initially, a small loss of 3.0% was detected within a temperature range of 100 °C to 198.95 °C. During the second stage, a loss of 96.3% of the mass was detected in the interval of 198.95 °C to 251.16 °C. The TG of the formulations showed three stages of decomposition, with an initial mass loss of 4.0% up to 200 °C during the first stage. An increase in temperature, in the range of 250 °C to 350 °C, caused a degradation of 18.60% for WG and between 14.24% to 16.39% for PG2G, PG5G, and PG10G. The most significant mass loss (above 78%) in the gels was observed in the third event, in the range of 300 °C to 490 °C (Figure 2). The peak area of the DTG was directly proportional to the mass variation in the samples, thus demonstrating the agreement of results in the analysis of TG and DTG curves in all gel groups.

#### 2.2.3. Differential Scanning Calorimetry (DSC)

**Figure 3:**
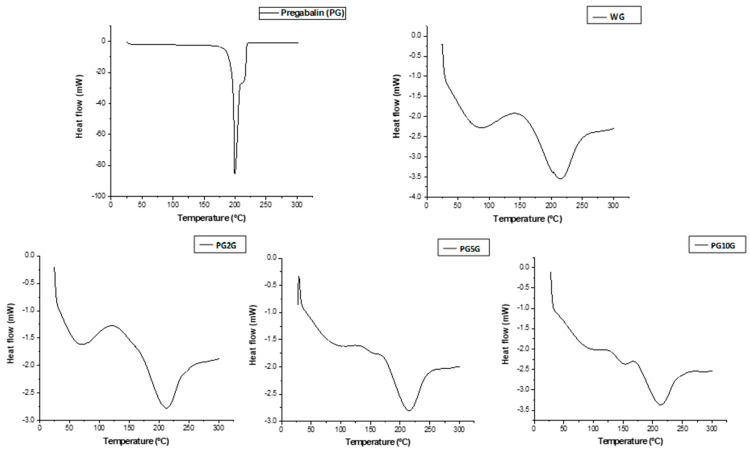
Differential Scanning Calorimetry (DSC) analysis of pregabalin (PG) and formulations, namely, WG (1.0% carbopol white gel), PG2G (2.0% pregabalin gel), PG5G (5.0% pregabalin gel), and PG10G (10% pregabalin gel).

The DSC of PG revealed a single endothermic peak, occurring within the temperature range between 196.90 °C to 210.03 °C. For the gels, a broad endothermic peak was observed at 50–97 °C in the WG and PG2G groups and was less pronounced in the PG5G and PG10G groups. An exothermic peak in the temperature range of 100 to 138 °C and a pronounced endothermic peak from 198 to 230 °C were present in all formulations (Figure 3).

### 2.3. Evaluation of Gel Formulation Stability

All formulations showed good stability. No phase separations were observed, both in the initial centrifugation test and after being subjected to experimental conditions during the stability studies (preliminary and accelerated). The gels maintained an average pH of 6, which remained stable throughout all evaluations. The formulations were analyzed for their appearance, color, and odor (Table 1). All of them exhibited a homogeneous appearance, with no changes in color or odor, during all cycles of the stability test. Additionally, no precipitates were formed under the conditions they were subjected to at the end of the 90-day study. 

### 2.4. Rheological Properties

**Figure 4:**
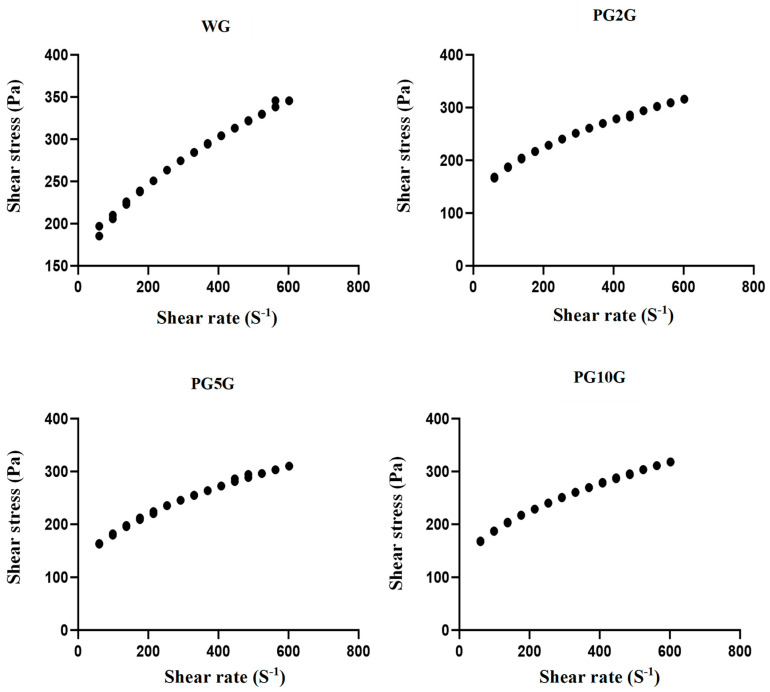
Viscosity profile of the different formulations: WG (1.0% carbopol white gel), PG2G (2.0% pregabalin gel), PG5G (5.0% pregabalin gel), and PG10G (10% pregabalin gel).

The 1.0% carbopol base gel exhibited a viscosity of 3310 cP, and after the incorporation of pregabalin solutions, the viscosity was reduced to 3000 cP in all formulations. However, there was no significant difference between the various concentrations of pregabalin present in the gels. Apparent viscosity decreased as the shear rate and shear time increased, both in the WG group and in PG2G, PG5G, and PG10G (Figure 4).

### 2.5. Evaluation of Cytotoxicity of Gel Formulations

#### Cell Viability Assessment

**Figure 5:**
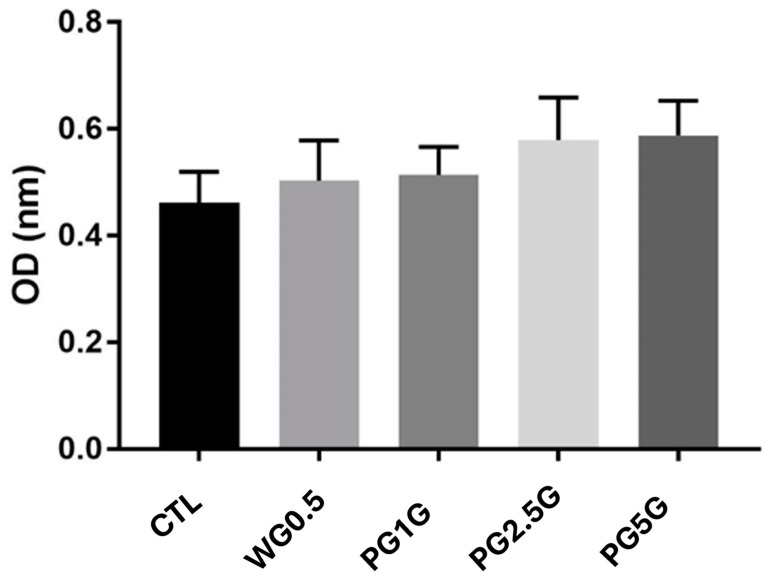
Cytotoxicity of different formulations after dilution in DMEM: CTL (control), WG (white carbopol gel 1.0%), PG1G (pregabalin gel 1.0%), PG2.0G (pregabalin gel 2.0%), and PG5G (pregabalin gel 5%).

As shown in Figure 5, the formulations did not induce cell death 24 h after cell seeding, and no statistically significant differences were observed between the analyzed groups (*p* > 0.05). Therefore, pregabalin gels did not affect cell viability, regardless of the drug concentration used.

## 3. Discussion

Investigations related to new protocols and therapeutic alternatives for the topical application of analgesic and anti-inflammatory drugs in the oral cavity are of great value to dentistry [21,22,23,24,25]. In this sense, pregabalin ((S)-2-(aminomethyl)-5-methylhexanoic acid) has been highlighted for its high efficacy in neuropathic pain models [4,26,27]. Used as an anticonvulsant, analgesic, and anti-inflammatory medication, its action and efficiency have led to the advent of the search for new formulations for use through different routes of administration, such as the topical route [14,17,18].

There are still no commercially available topical formulations containing pregabalin, but many studies are being conducted to make this administration alternative viable [13,15,28]. Because the oral route culminates in a series of undesirable and uncomfortable adverse reactions, topical formulations tend to reduce systemic drug exposure, minimizing the occurrence of CNS-mediated side effects [18]. Thus, this study aimed to verify the properties and characteristics exhibited by PG gels to ensure their quality standard and subsequent safety for topical application in the oral cavity. The concentrations used were based on previous studies [18], resulting in transparent pregabalin gels with a pH of 6 and suitable consistency for application to the oral mucosa.

Gel formulations are widely used as a vehicle for the topical application of medications [29,30]. These pharmaceutical forms are composed of an aqueous part that is retained in a polymeric matrix, which is composed of gelling substances that evaporate over time, leaving an adhesive film on the contact area [31]. Topical analgesic formulations such as gels, unlike systemic medications, act only at the site of contact, thereby enhancing drug absorption into the desired tissue [32]. Moreover, they are considered less aggressive than formulations that require ingestion (leading to possible adverse reactions) and possess potent, safe, and effective analgesic effects [31,33].

In this study, different concentrations of the drug content were analyzed to find possible influences on the formulation composition. Nagao et al. [34] observed that due to pregabalin’s high solubility in water, the maximum concentration in an aqueous solution is 12 mg/mL (1.2% *w*/*v*) at 25 °C. Additionally, for the incorporation of pregabalin into gel and cream bases, dilution followed by centrifugation at a low temperature (4 °C) was necessary [34]. This process promotes phase separation and allows for the use of the supernatant containing desirable drug concentrations. Therefore, the concentrations used in the gels obtained in this study took into account concentrations that allowed for the incorporation of the drug’s aqueous solution into the carbopol base gel and showed efficacy when evaluated in in vivo pain models [14,16,17].

One of the techniques used in this research to characterize and investigate the composition of the drug and the formulations obtained was Fourier-transform infrared spectroscopy (FTIR). This method identifies the characteristic functional groups of the analyzed organic compounds, providing a preliminary study of their chemical structure, both for isolated substances and complex samples [35]. The FTIR spectra of pregabalin found in this study are in agreement with previous research [14,36]. Regarding the base gel and pregabalin gels, the absorption bands found may be related to the excipient used, also suggesting the presence of pregabalin in the formulation, with a peak similar to those observed in other studies [37,38]. However, since the gel and pregabalin have peaks at the same wavelength, the peak of pregabalin does not appear clearly when analyzing the gel loaded with the drug. This may be due to pregabalin being soluble and homogeneously dispersed in the gel matrix, and since FTIR is not a specific method, it cannot distinguish pregabalin when it is in the gel. Therefore, the occurrence of interactions in the solid state between drugs and excipients in solid pharmaceutical forms can lead to changes in stability, solubility, dissolution, and drug bioavailability [39]. The technique of Differential Scanning Calorimetry (DSC) combined with TG/DTG techniques has been shown to be very useful in pre-formulation studies for investigating and predicting physicochemical incompatibilities between drugs and excipients [40]. For pregabalin, decomposition events are observed at temperatures ranging from approximately 168 °C to 275 °C [36], a result similar to that found in the present study. High and narrow peaks are characteristic of a crystalline substance, as reported by Steendam et al., 2019 [41]. The hydrated form of pregabalin is physically unstable because it quickly converts back to its anhydrous form, a fact that led to the investigation of its thermal properties by DSC for both the pure drug and the gels.

Regarding the gel samples (WG, PG2G, PG5G, and PG10G), equivalent thermal events were observed, in addition to the characteristic initial mass loss, which generally occurs due to the presence of water in the composition of semi-solid formulations and its subsequent evaporation [42,43]. Thus, the different concentrations of pregabalin in the formulations do not seem to influence the thermal characteristics of the product. It is worth noting that thermogravimetry is a technique that assists in the determination of purity, moisture content, and thermal stability of the material and ensures that it remains active, effective, and safe for use after formulation [44].

Furthermore, the crystalline nature of pregabalin generates an endothermic event, which can be observed in DSC analyses, related to the substance’s melting [36]. Pregabalin exhibited an endothermic peak, consistent with other studies [36,45]. For the gels, the broad peak observed may indicate the evaporation of water adsorbed by carbopol [37]. Moreover, the results obtained may reveal that the incorporation of pregabalin into the gel tends to alter it from a more crystalline to a less crystalline/amorphous form [28,43].

A previous study evaluated different preparations of pregabalin for topical transdermal application [17]. In it, the organogel formulation showed better results, such as higher permeability and significant analgesic effect. Arafa and Ayoub [16] also evaluated mucoadhesive topical gels with pregabalin isolated or encapsulated in niosomes, aiming to analyze different drug release forms, and observed that formulations of HPMC and carbopol hydrogel showed a higher percentage of PG release compared to niosomes. Therefore, the pharmaceutical form for drug delivery directly influences characteristics related to its action and release.

The lower percentage of release found in the study by Arafa and Ayoub [16] regarding PG gels with carbopol is related to the higher viscosity of this gel base compared to HPMC gels. Additionally, carbopol has a faster rate of polymer swelling and relaxation [46]. However, the consistency obtained in the present study for the carbopol 940 gel was adequate for application in the oral cavity. Furthermore, it was observed that the different concentrations of the drug do not seem to influence the thermal characteristics of the formulation, indicating no interaction between the components of the 1.0% carbopol base gel with pregabalin. 

To determine the influence of different formulations on the action of PG, Haddad and Hasian [47] evaluated different emulgel formulas containing pregabalin using carbopol 940 and other polymers. The best percentage of drug release was achieved with carbopol 940 and HPMC, offering rapid analgesic effects and avoiding pregabalin-mediated side effects in the central nervous system. Thus, optimized gel formulations containing pregabalin resulted in a significant release rate of up to 90% and better dermal permeation [31].

Regarding carbopol gels, their rheological behavior is commonly studied [42]. This type of analysis is important as it is directly related to physical properties, adhesion characteristics, residence time in the application area, and the diffusion and release of the active ingredient through the gel’s microstructure [48,49]. Therefore, rheological characteristics are important properties that should be considered in the manufacturing, storage, and application processes of a product [50]. Thus, rheological measurements were useful for characterizing the viscoelastic properties of pregabalin gels.

Semi-solid pharmaceutical forms, for the most part, exhibit the property of adhesion to the application surface for a certain period before being removed. This behavior allows them to maintain their shape and stay in place until an external force causes them to deform and flow [51]. Thus, it is desirable for topical products to spread easily without being fluid enough to run off the surface. In the present study, high-viscosity gels were obtained, which, when subjected to an increase in deformation rate and shear, had lower viscosity. This occurs due to the orientation of molecules in the direction of the flow and the breaking of aggregates, which makes the resistance to movement progressively lower [48].

The formulations obtained showed good stability, regardless of the concentration of pregabalin used. Therefore, stability studies provide information indicating the degree of the relative stability of a product under various conditions from its manufacture to the end of its shelf life [48]. This stability is considered relative because it varies over time and depends on factors that accelerate or retard changes in the product’s parameters [39,52]. Tests should be conducted under conditions that provide information on the product’s stability in the shortest possible time, as conducted in this study.

Despite being widely used to treat acute and chronic neuropathic pain [4,6,11,13,53,54], it is still unclear whether pregabalin has harmful properties in oral mucosa cells. Therefore, the evaluation of cytotoxicity is a necessary step in assessing the biocompatibility of pharmaceutical forms. In this study, gels with different concentrations of the drug were used in a human gingival fibroblast cell line to elucidate this issue. None of the formulations caused cell death. Thus, pregabalin gels did not affect cell viability, regardless of the concentration of the drug used, indicating they were not cytotoxic to the cells used. This is consistent with the limited evidence provided so far by other studies that investigated the potentially harmful effects of pregabalin on other cellular models [15,36,55].

In this context, therapeutic materials during their clinical use should have a desirable action, maintain maximal tissue vitality, and at the same time, have insignificant or null cytotoxic effects, causing no potential damage to target cells [56]. Thus, in vitro cytotoxic screening as a primary factor of biocompatibility is determined by cell culture, and the MTT assay is one of the most widely used tests to evaluate the cytotoxicity of materials of different origins in cell cultures [57]. It is recommended that cells of potential clinical relevance be selected for in vitro toxicity testing [56]. Therefore, the present assay was performed on human gingival fibroblasts.

Characteristics such as stability and biocompatibility are paramount in the investigation of new pharmaceutical forms for drug delivery [39]. Typical problems encountered during the preparation of new forms are identified in the pre-formulation stage, which evaluates factors such as insufficient mixing, segregation, and interaction between components [47]. Thus, the correct choice of excipients with their appropriate functional role, as well as good compatibility between them, provides content uniformity, proper drug distribution, and safety for their application [32].

## 4. Materials and Methods

### 4.1. Materials

Carbopol 940 was purchased from Mapric (São Paulo, Brazil), propylene glycol ≥100% was purchased from Viafarma (São Paulo, Brazil), methylparaben M, ≥100% was purchased from VPK farma (São Paulo, Brazil), propylparaben M, ≥100% was purchased from Fagron Brasil (São Paulo, Brazil), triethanolamine P.A, ≥99.5% was purchased from LabSynth (São Paulo, Brazil), ethylenediaminetetraacetic acid P.A. A.C.S (P.M 372.24) (EDTA) was purchased from Quimibrás Indústrias Química S.A (Rio de Janeiro, Brazil), Dulbecco’s Modified Eagle Medium (DMEM) and 3-(4,5-dimethylthiazol-2-yl)-2,5-diphenyl tetrazolium bromide ≥98% (MTT) were purchased from Sigma-Aldrich (Saint Louis, MO, USA), and Dimethylsulfoxide P.A. ACS, ≥99% (DMSO) was purchased from Neon (São Paulo, Brazil). The pregabalin (≥98.0–102.0%) used in this study was purchased from Vardhman (Chemtech Ltd., Punjab, India) and was in the form of a white crystalline powder.

### 4.2. Obtaining the Gel Formulation

The carbopol 940 gel base was used as a vehicle for delivering the active pharmaceutical ingredient, i.e., pregabalin (PG). The gel base composition (Table 2) consisted of carbopol 940, propylene glycol, methylparaben, propylparaben, and distilled water. The excipients were individually weighed on an analytical balance and transferred to a 500 mL beaker, where they were left to hydrate for 24 h. After the resting period, the system was homogenized using a mechanical stirrer (Fisatom 713 DS, São Paulo, Brazil) at 1000 rpm for 10 min, gradually adding triethanolamine to adjust the pH to 6, thus forming the non-solid formulation, i.e., the gel base, which was then placed in hermetically sealed containers. Subsequently, to obtain the pregabalin gels, different concentrations of the pregabalin solution (Table 2) were incorporated into the gel base.

### 4.3. Preparation of Pregabalin Solutions 

The PG solutions were prepared by diluting the drug in ultrapure water to concentrations of 2.0, 5.0, and 10.0 mg/mL (Table 2). The solution was then agitated for 10 min in an ultrasonic bath (Cleaner Kondentech^®^ CD-4820, São Paulo, Brazil) and centrifuged at 4 °C for 10 min. After centrifugation, the supernatant (clear liquid portion) was collected and subsequently incorporated into the 1.0% carbopol gel base [17].

### 4.4. Characterization of the Drug and Gel Formulation

#### 4.4.1. Fourier-Transform Infrared Spectrometry (FTIR)

The FTIR spectra of pregabalin and the formulations were obtained using Fourier-transform infrared (FTIR) absorption spectroscopy on an IR Prestige 21 spectrometer (Shimadzu^®^, Kyoto, Japan). For the gel, the attenuated total reflection (ATR) technique was used, while for pregabalin, the sample was prepared as a KBr pellet (0.01 g of pregabalin mixed with 0.1 g of KBr powder). All samples were scanned with 32 scans in the absorption range of 4000–500 cm^−1^ with a resolution of 4 cm^−1^ [45].

#### 4.4.2. Thermogravimetric Analysis (TG/DTG)

The thermogravimetric curves (TG/DTG) of pregabalin and the gel formulations were obtained using a TGA 50 thermal analyzer (Shimadzu^®^, Kyoto, Japan). Briefly, 8.0 mg of each sample was weighed and transferred to a platinum crucible. Subsequently, they were subjected to a temperature range of 25 °C to 600 °C under a dynamic nitrogen atmosphere (50 mL/min) and a heating rate of 10 °C/min. The calculations of mass loss were performed using the TA-60W software version 2.21 (Shimadzu^®^, Kyoto, Japan) [36].

#### 4.4.3. Differential Scanning Calorimetry (DSC)

To obtain the DSC curves, the DSC-60 plus equipment (Shimadzu^®^, Kyoto, Japan) was employed. Each sample (8.0 mg) was weighed and transferred to an aluminum crucible. Subsequently, they were subjected to a temperature range of 25 °C to 300 °C under a dynamic nitrogen atmosphere (50 mL/min) and a heating rate of 10 °C/min. The calculations of enthalpy difference (∆H) were performed using the TA-60W software version 2.21 (Shimadzu^®^, Kyoto, Japan) [45].

### 4.5. Evaluation of Gel Formulation Stability

#### 4.5.1. Centrifugation Test

The centrifugation test was conducted in triplicate, using 5.0 mg of the samples weighed in Falcon tubes and placed in a centrifuge (Eppendorf 5804 R, Hamburg, Germany) under the following experimental conditions: temperature of 25 °C ± 2.0 °C; rotation speed of 3000 rpm; and test duration of 30 min [52].

#### 4.5.2. Preliminary Stability Study

In the stability study, each sample (5.0 g) was placed in neutral transparent glass vials (n = 10) and subjected to thermal stress. The vials were properly labeled and filled with 2/3 of their volume with the samples, allowing for gas exchange. The formulations, in triplicate, were divided into four groups: refrigerator (5 °C), oven (40 °C), freeze/thaw cycle (5 °C and 40 °C), and room temperature (27 °C). The samples belonging to the freeze/thaw cycle group were subjected to temperature stress conditions to accelerate the appearance of possible signs of instability. The remaining samples remained at the same temperature (refrigerator, oven, and room temperature) throughout the evaluation period, which was 15 days in total. The adopted values for elevated temperatures were T = 40 ± 2 °C (oven), and for low temperatures, T = 5 ± 2 °C (refrigerator). In the freeze/thaw groups, cycles of 48 h were performed, with 24 h at 40 ± 2 °C and 24 h at 5 ± 2 °C, over the 15 days (6 cycles). The samples were evaluated at the end of the 1st, 4th, and 6th cycles. The groups that remained in the refrigerator, oven, and room temperature were evaluated at T0 (immediately after preparation), T24h (after 24 h), T7 (7 days), and T15 (15 days) [52].

#### 4.5.3. Accelerated Stability Study

In the accelerated stability study, the samples in triplicate were stored in an oven (±40 °C), refrigerator (±5 °C), or at room temperature (27 °C) for 90 days. The analyses were conducted at time points T0 (immediately after preparation), T7 (7 days), T15 (15 days), T30 (30 days), T60 (60 days), and T90 (90 days). After each cycle, the samples were evaluated for pH value and organoleptic characteristics (emphasizing possible changes in appearance, color, and odor). Homogeneity, brightness, and absence of lumps and precipitates were also analyzed [52].

### 4.6. pH Value Determination

The pH of the gel formulations was determined using the pH meter MB-10 micro (Marte Científica, São Paulo, Brazil) previously calibrated with buffer solutions (pH of 4.0 and 7.0). Readings for each sample were taken through three determinations [52].

### 4.7. Rheological Properties

The viscosity of the gels was determined using a Brookfield rheometer and a Brookfield RST+RHEOMETER (Brookfield, MA, USA) with spindle number RCT-50-1, rotating at 100.01 rpm at a fixed temperature (36.17 °C) for a duration of 120 s. The rheological behavior of the samples was performed in triplicate [16].

### 4.8. Evaluation of Cytotoxicity of Gel Formulations

#### 4.8.1. Preparation of Gel Formulations

In the preparation of solutions for cell incubation, 1 mg of each formulation was weighed: WG (1.0% carbopol gel base/white), PG2G (2.0% pregabalin gel), PG5G (5.0% pregabalin gel), and PG10G (10% pregabalin gel). These were diluted in 1 mL of DMEM (Sigma, St. Louis, MO, USA) so that the final concentration was 1 mg/mL. Therefore, the final concentrations were WG (0.5% white carbopol gel), PG1G (1.0% pregabalin gel), PG2.0G (2.0% pregabalin gel), and PG5G (5% pregabalin gel), respectively.

#### 4.8.2. Cell Culture and Maintenance

The human immortalized gingival fibroblast was kindly provided by the Cell Culture Laboratory of the Faculty of Dentistry (UFPA, Belém, Brazil) [58] and cultured at a density of 5 × 10^4^ cells/cm^2^ in DMEM medium supplemented with 10% fetal bovine serum (Gibco, Carlsbad, CA, USA). It was maintained in an incubator at 37 °C under a 5.0% CO_2_ atmosphere until it reached 80% confluence for further use.

#### 4.8.3. Cell Viability Assessment

In 24-well plates, 1 × 10^5^ cells/mL were added per well. The plates containing the cells were then incubated for 24 h, together with WG, PG1G, PG2.5G, and PG5G, as described previously. After this period, the supernatant was removed, and 0.5 mg/mL of MTT ([3-(4,5-dimethylthiazol-2-yl)-2,5-diphenyl tetrazolium bromide—Sigma^®^) diluted in PBS was added and incubated for 3 h. Then, the supernatant was removed again, and 200 μL of DMSO was added to each well to solubilize the formazan crystals. The plates were then agitated for 10 min. The resulting solution was read in a spectrophotometer (BIO-RAD Model 450 Microplate Reader, São Paulo, Brazil) at a wavelength of 570 nm [58]. As a control for the assay, cells were killed with a 10% formalin solution in PBS, and as a control for cell viability, another group of cells was cultured without the presence of the gels. The result was expressed considering the optical density obtained (OD-570 nm). Three independent experiments in triplicate were conducted for each gel concentration.

### 4.9. Statistical Analysis

The cell viability data were analyzed using GraphPad Prism 6™ software (version 10.2.0), employing the Student’s *t*-test. A statistical difference was considered significant for *p*-values < 0.05.

## 5. Conclusions

The pregabalin gels for topical application in the oral cavity were developed in this study. Physicochemical characterization was performed to control the quality of the formulations at different concentrations of pregabalin (2.0%, 5.0%, and 10%), confirming the presence of the drug’s functional groups in the prepared gels, as well as the thermal stability of the product. Preliminary and accelerated stability studies, which added safety to the evaluated formulations over time, demonstrated that the product maintains favorable characteristics and stability throughout the analyzed period.

Rheological analysis revealed a profile of a semi-solid dosage form, ideal for the proposed application in this study. A cytotoxicity evaluation indicated that pregabalin gels do not present toxicity to human gingival fibroblasts, suggesting their suitability for formulations intended for application on the oral mucosa. Thus, pregabalin gels have been shown to be safe, with satisfactory quality, ensuring the effectiveness of the developed formulation. Even the incorporation of larger quantities of the drug (10%) did not reveal impairments to the formulation, making it a viable option for use. Therefore, this product may represent a promising therapeutic alternative for the topical application of this medication in the oral cavity.

## Figures and Tables

**Table 1:** Results of pH (mean and standard deviation) and organoleptic characteristics evaluated in different gels.

Characteristics	Formulation			
	WG	PG2G	PG5G	PG10G
Aspect	Ho	Ho	Ho	Ho
Color	I	I	I	I
Odor	C	C	C	C
pH	5.48 ± 0.10	6.07 ± 0.07	6.15 ± 0.09	6.33 ± 0.17

Legend: Ho—homogeneous, I—colorless, and C—characteristic.

**Table 2:** Composition and quantity of components in the formulations.

Components	Quantity (%)			
	Gel W (Gel Base/White)	Gel PG2.0	Gel PG5.0	Gel PG10
Carbopol 940	1	1	1	1
Methylparaben	0.01	0.01	0.01	0.01
Propylparaben	0.05	0.05	0.05	0.05
EDTA	0.1	0.1	0.1	0.1
Propylene glycol	3	3	3	3
Triethanolamine 50% (*v/v*)	qs	qs	qs	qs
Pregabalin	-	2	5	10
Distilled water	qsp 100	qsp 100	qsp 100	qsp 100

Note: qsp: quantity sufficient to; qs: quantity sufficient; and EDTA: ethylenediaminetetraacetic acid.

## Data Availability

Additional research data can be requested directly from the corresponding author.
